# Weight-for-length, early weight-gain velocity and atopic dermatitis in infancy and at two years of age: a cohort study

**DOI:** 10.1186/s12887-017-0889-6

**Published:** 2017-06-07

**Authors:** Teresa Løvold Berents, Karin Cecilie Lødrup Carlsen, Petter Mowinckel, Håvard Ove Skjerven, Leif Bjarte Rolfsjord, Live Solveig Nordhagen, Bente Kvenshagen, Jon Olav Gjengstø Hunderi, Maria Bradley, Per Medbøe Thorsby, Kai-Håkon Carlsen, Petter Gjersvik

**Affiliations:** 1 Institute of Clinical Medicine, University of Oslo, Oslo, Norway; 2 Department of Dermatology, Oslo University Hospital, Oslo, Norway; 3 Department of Paediatrics, Oslo University Hospital, Oslo, Norway; 4 Department of Paediatrics, Innlandet Hospital, Oslo, Norway; 5 Diakonova University College, Oslo, Norway; 6 Department of Paediatrics, Østfold Hospital, Grålum, Norway; 7 Department of Molecular Medicine, Karolinska Institutet at Karolinska University Hospital, Solna, Sweden; 8 Hormone Laboratory, Department of Medical Biochemistry, Oslo University Hospital, Oslo, Norway

**Keywords:** Overweight, Weight-for-length, Infancy, Atopic dermatitis

## Abstract

**Background:**

Overweight and atopic dermatitis (AD) are major health problems in most industrialised countries, but the relationship between overweight and AD in infants and young children is unclear. We investigated if weight-for-length at birth, in infancy and at two years, as well as early weight-gain velocity, are associated with the development of AD in early life.

**Methods:**

Cohort study of infants (*n* = 642), all living in south-east Norway, hospitalized with acute bronchiolitis (*n* = 404) or recruited from the general population (*n* = 238), examined at mean age 5.1 months (enrolment) and at a two-year follow-up visit (*n* = 499; 78%) at mean age 24.6 months. Exposures were weight-for-length (g/cm) at birth, enrolment and two-year follow-up, and early weight-gain velocity (gram/month from birth to enrolment). Excessive weight-for-length was defined as weight-for-length >95^th^ percentile of WHO child-growth standards. Data on weight-for-length at the three time points were obtained for 435, 428 and 473 children. AD was diagnosed according to the Hanifin & Rajka criteria or from a history of physician-diagnosed AD. We performed multivariate analyses with weight-for-length at birth, at enrolment and at the two-year follow-up visit and with early weight gain velocity for the endpoint AD at each visit.

**Results:**

In adjusted analyses, excessive weight-for-length at enrolment was associated with concurrent AD (OR 3.03; 95% CI 1.23–7.50) and with AD at two years (OR 2.40; 1.11–5.17). In infants without AD, weight-for-length at enrolment increased the risk of AD at two years, with OR being 1.02 (95% CI 1.00–1.04) per increased gram/cm. AD at two years was not associated with concurrent excessive weight-for-length, nor was AD at any time associated with weight-for-length at birth or with early weight-gain velocity.

**Conclusions:**

The results suggest that overweight in infancy may contribute to the development of AD in early life, highlighting the need for child health-care professionals to address potential overweight and atopic disease when advising infants’ caregivers.

**Trial registration:**

ClinicalTrials.gov number, NCT00817466, EudraCT number, 2009–012667-34.

## Background

Overweight and obesity are major health problems in most industrialised countries [1]. In some studies, overweight and obesity in children, adolescents and adults have been shown to be associated with atopic dermatitis (AD) [2], a chronic inflammatory skin disease, characterized by skin barrier and immunological dysfunction [3]. Also, overweight and obesity in children and adults without AD have been associated with skin barrier dysfunction [4, 5] and altered immunological responses [6]. The prevalence of AD has increased during the last 20–30 years, especially in young children [3], partly overlapping the increase in prevalence of obesity [7]. With the complex aetiology of AD, involving both genetic factors, such as *filaggrin* (*FLG*) mutations, and environmental factors [3, 8], the increased prevalence of obesity and overweight in early childhood could contribute to the AD epidemic seen in children [6].

In the present study, our main aim was to investigate if excessive weight-for-length at birth, in infancy or in early childhood is associated with the development of AD in the first years of life. Also, we aimed to investigate if early weight-gain velocity is associated with AD, and if *FLG* mutations may have an impact on possible associations.

## Methods

### Design

**Fig. 1:**
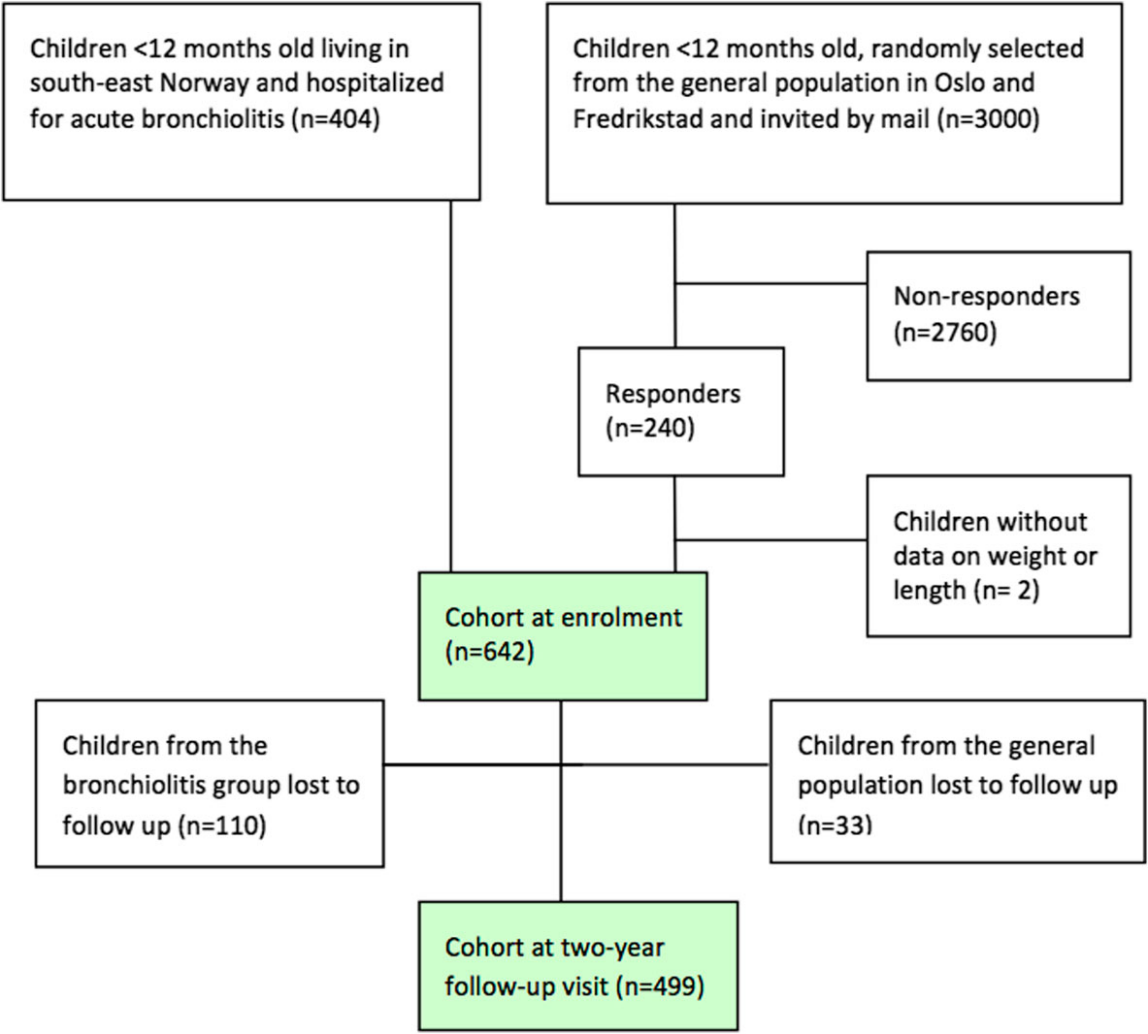
Flow chart of study cohort with 404 children hospitalized for acute bronchiolitis and 238 children recruited from the general population. The two-year follow-up visit was attended by 294 children from the bronchiolitis group (73%) and 205 (85%) of the children recruited from the general population

In this cohort study, infants living in south-east Norway were recruited through either being enrolled during hospital admission for acute bronchiolitis (*n* = 404) to a randomized clinical trial on airway obstruction treatment at eight hospitals in 2010–14 [9, 10] or as controls (*n* = 240) of similar age invited by letter sent to caregivers of 3000 infants from a general population in south-east Norway (Fig. 1) [11, 12]. Inclusion criteria for the bronchiolitis trial was moderate to severe acute bronchiolitis leading to hospitalization before 12 months of age, excluding those having received any glucocorticoid therapy in the preceding four weeks. The inclusion criterion for the controls was age 0–12 months of age at time of invitation. Exclusion criteria were serious cardiac, immunologic, neurologic, oncologic or pulmonary disease other than bronchiolitis. Study participants were invited later by letter and/or phone call to their caregiver(s) to attend a two-year follow-up visit 18 months after enrolment.

Infants were investigated at enrolment (*n* = 644) and at the two-year follow-up visit (*n* = 499) with a follow-up rate of 73% in the bronchiolitis group and 85% in the general population group. Characteristics from birth were obtained through structured parental interviews and children’s health cards. Investigations included general clinical and specific skin assessment, weight and length measures, blood sampling and transepidermal water loss (TEWL) measurements (at enrolment only in children from general population).

Caregivers for all infants were informed orally and in writing, and informed written consents were obtained from caregivers for all infants. The Regional Committee for Medical and Health Research Ethics South East Norway approved the study. The biobank was registered according to current regulations and the bronchiolitis trial was registered at ClinicalTrials.gov number, NCT00817466, EudraCT number, 2009–012667-34.

### Subjects

From the original cohort of 644 infants, weight and/or length were recorded at birth, at enrolment and/or at the two-year follow-up visit in 642 children (Fig. 1). Mean age (min, max) was 5.1 months (0.2, 13.4) at enrolment and 24.6 months (17.5, 35.2) at the two-year follow-up visit.

### Clinical examination and measurements

Structured interviews with caregivers were performed addressing previous and current health of the child and the family members, parental socio-economic factors and ethnicity, duration of exclusive breastfeeding, duration of breastfeeding, and parental atopy. Weight (in grams) and length (in centimetres) were measured by trained nurses with the infant undressed in a supine position at the enrolment examination and supine or standing position at the two-year follow-up visit. Data on weight and length at birth were obtained from the infants’ health cards and/or reported by the caregiver. In both groups, AD was diagnosed clinically by experienced physicians based on the diagnostic criteria of Hanifin & Rajka [13] or on a caregivers’ history of physician-diagnosed AD. Severity of AD was assessed at both visits using the SCORing Atopic Dermatitis index (SCORAD index) [14], reported as the mean of assessments by two trained investigators.

Data on the four most common *FLG* mutations in the European population, i.e. R501X, 2282del4, R2447X and S3247X were obtained in 558 children [12]. Data on vitamin D levels in the children at enrolment and at two years were obtained for 595 and 450 children, respectively [12]. TEWL was measured on non-lesional skin on the lateral part of upper arm, using the open chamber DermaLab USB (Cortex, Hadsund, Denmark) system and accepting ambient temperatures at 20–25 °C and ambient humidity at 20–50% [11]. TEWL values were reported as the mean of three measurements.

### Outcomes, exposure and confounding variables

The main outcomes were AD at enrolment and at the two-year follow-up visit. The main exposure variables were weight-for-length at birth, at enrolment and at the two-year follow-up visit, early weight-gain velocity and body mass index (BMI). Weight-for-length, calculated as the ratio between weight (g) and length (cm), was used as a bivariate exposure variable of excessive weight-for-length >95^th^ percentile according to World Health Organization (WHO) Child Growth Standards [15] versus all other, as well as a continuous variable with each unit representing gram/cm. Early weight-gain velocity was defined as weight gain (in grams) per month from birth to age at enrolment. Body mass index was defined as weight (in kilograms) divided by the square of the length/height (in meters).

Potential confounding variables were chosen on the basis of known or possible associations with AD and/or weight-for-length, such as age, sex, gestational age, being firstborn, parental atopy, income, education and ethnicity, duration of exclusive breastfeeding, duration of breastfeeding, and vitamin D levels, as well as recruitment source, i.e. bronchiolitis group or general population.

### Statistical analyses

Data are presented as number and percentages, except for continuous data, which are presented as means with standard deviation (SD), min-max or 95% confidence intervals (CI). Pearson’s chi-square test was used for analyses of categorical data, while independent sample t-test was used for continuous variables. WHO Child Growth Standards igrowup package [15] was used to calculate z-scores for weight-for-length. Z-scores >1.64 was defined as >95^th^ percentile.

We performed multivariate analyses with weight-for-length at birth, at enrolment and at the two-year follow-up visit and with early weight-gain velocity for the endpoint AD at each visit or at either visit. Multivariate logistic regression analyses in the final models included all variables with a *p*-value <0.25 in bivariate analyses. The Hosmer’s step down procedure [16] was performed and repeated until all factors were significant at a level of *p* < 0.05. Weight-for-length and early weight-gain velocity were retained in the multivariate models even when non-significant. All analyses were repeated using BMI instead of weight-for-length. The final models were checked for confounding and interactions with the variables previously mentioned, and for interaction with *FLG* mutation. Because of non-normality, the analysis of the association between TEWL and weight-for-length as continuous variables was assessed by robust regression analysis [17].

Missing data were verified being completely missing at random by the use of Little’s test [18] with no imputation of missing data. Statistical power calculation was performed post hoc based on the assumption that the prevalence of AD was 11% in the first year of life [12]. A population size of 499 children would give a statistical power of 68% to detect at least an 4% increase in AD prevalence for one unit increase in weight-for-length and BMI, assuming R^2^ of 0.10. The level of statistical significance was set to 0.05.

## Results

Clinical and background characteristics are presented in Table 1. The children from the bronchiolitis group differed in some respects from the those from the general population (Table 1). Data on weight and length were obtained from 435 children at birth, 428 at enrolment and 473 at the two-year follow-up visit (Table 2). At enrolment, AD was diagnosed in 55 of 428 children (13%), of whom 41 (75%) fulfilled Hanifin & Rajka’s diagnostic criteria [13]. At the two-year follow-up visit, AD was diagnosed in 106 of 473 children (22%), of whom 72 (68%) fulfilled Hanifin & Rajka’s diagnostic criteria (Table 2).

**Table 1:** Demographic and clinical characteristics of 642 infants included in the cohort study. Numbers in first columns of each group specify the number of infants with obtained data

	Recruited from bronchiolitis trial (*N* = 404)			Recruited from the general population (*N* = 238)			
	No.			No.			*P* value
Male sex, No. (%)	404	240	(59)	238	133	(56)	.2
Age at enrolment, mean (min, max), months	404	4.2	(0.2, 12.0)	238	6.6	(1.0, 13.4)	<.001
Age at two-year follow-up visit, mean (min, max), months	294	24.6	(19.0, 35.2)	205	24.5	(17.5, 35.2)	.83
Father Caucasian, No. (%)	287	266	(93)	199	184	(93)	.53
Mother Caucasian, No. (%)	289	263	(91)	202	192	(95)	.06
High parental education,^a^ No. (%)	351	245	(70)	238	219	(92)	<.001
Low parental income,^b^ No. (%)	294	26	(9)	203	5	(3)	.002
Firstborn child, No. (%)	294	66	(22)	205	101	(61)	<.001
One or more sibling living at home, No. (%)	289	251	(87)	200	118	(59)	<.001
Pet ownership,^c^ No. (%)	281	85	(30)	201	46	(23)	.05
Parental atopy,^d^ No. (%)	293	213	(73)	205	152	(74)	.40
Maternal atopy	293	150	(51)	205	112	(55)	.25
Paternal atopy	293	120	(41)	205	86	(42)	.45
Exposed to in-door smoking at home, No. (%)	293	7	(2)	199	1	(0.5)	.10
Gestational age, mean (min, max), weeks	302	38.7	(26, 42)	223	39.6	(28, 42)	<.001
Exclusive breastfeeding duration, mean (SD), months	278	4.5	(2.4)	193	4.8	(2.2)	.14
Any breastfeeding, mean (SD), months	280	8.6	(5.3)	193	10.6	(5.6)	<.001
Vitamin D level at enrolment, mean (min, max), nmol/l	366	52.9	(6.0, 132.0)	229	66.5	(6.0, 120.5)	<.001
Vitamin D level two-year follow-up visit, mean (min, max), nmol/l	259	66.3	(6.0, 142.9)	191	67.8	(16.6, 130.0)	.39
*Filaggrin* mutation,^e^ No. (%)	361	26	(7)	203	16	(8)	.44
TEWL^f^ at enrolment, median (Q1, Q3), g/m^2^/h	NA	NA	NA	165	8.20	(5.90, 10.43)	NA
TEWL^f^ at two-year follow-up visit, median (Q1, Q3), g/m^2^/h	204	5.98	(4.28, 9.20)	156	5.08	(3.28, 7.58)	.04

^a^One or both parents reporting education beyond 12 (or 13) years schooling

^b^< 500,000 NOK per year, approx 60,000 USD

^c^Having dog, cat, rabbit, hamster, guinea pig and/or parakeet

^d^Mother and/or father reporting asthma, allergic rhinitis, atopic dermatitis, food allergy and/or urticaria

^e^R501X, 2282del4, R2447X, S3247X, all heterozygeous

^f^Measured on lateral part of upper arm

*TEWL* transepidermal water loss ,*Q1* lower quartile, *Q3* upper quartile, *NA* not assessed

**Table 2:** Data on anthropometrics and atopic dermatitis in children recruited from bronchiolitis trial and from the general population. Numbers in first columns of each group specify number of infants with obtained data

	Recruited from bronchiolitis trial (*N* = 404)			Recruited from the general population (*N* = 238)			
	No.			No.			*P* value
Birth							
Weight, mean (SD), g	280	3392	(645)	201	3591	(558)	<.001
Length, mean (SD), cm	253	49.9	(3.0)	193	50.6	(2.3)	.002
Weight-for-length, mean (SD), g/cm	253	68.6	(10.2)	193	70.8	(9.1)	.02
z-score weight-for-length,^a^ mean (SD)	242^b^	0.21	(1.4)	193	0.24	(1.2)	.83
Excessive weight-for-length,^a,c^ No. (%)	242^b^	31	(13)	193	19	(10)	.23
BMI, mean (SD), kg/cm^2^	253	13.7	(1.8)	193	14.0	(1.5)	.15
Enrolment							
Weight, mean (SD), g	404	6510	(1874)	227	7829	(1769)	<.001
Early weight-gain velocity, mean (SD), g/month	280	767	(271)	190	737	(242)	.22
Length, mean (SD), cm	201	62.9	(7.2)	227	67.8	(6.4)	<.001
Weight-for-length, mean (SD), g/cm	201	104.8	(19.9)	227	114.3	(17.7)	<.001
z-score weight-for-length,^a^ mean (SD)	201	0.16	(1.6)	227	0.03	(1.2)	.35
Excessive weight-for-length,^a,c^ No. (%)	201	26	(13)	227	15	(7)	.02
BMI, mean (SD), kg/cm^2^	201	16.6	(2.2)	227	16.8	(1.8)	.30
Atopic dermatitis,^d^ No. (%)	201	14	(7)	227	41	(18)	<.001
SCORAD, median (IQR)	201	NA	NA	36	16	(11, 21)	NA
Two-year follow-up visit	294			205			
Weight, mean (SD), kg	274	13.2	(1.7)	201	12,9	(1.6)	.05
Length, mean (SD), cm	275	87.1	(4.1)	202	88.6	(4.4)	<.001
Weight-for-length, mean (SD), g/cm	273	151. 1	(15.5)	200	145.4	(14.7)	<.001
z-score weight-for-length,^a^ mean (SD)	273	1.10	(1.1)	200	0.54	(1.1)	<.001
Excessive weight-for-length,^a,c^ No. (%)	273	83	(30)	200	30	(15)	<.001
BMI, mean (SD), kg/cm^2^	273	17.3	(1.7)	200	16.4	(1.6)	<.001
Atopic dermatitis, No. (%)	273	59	(22)	200	47	(24)	.35
SCORAD, median (IQR)	44	13	(10, 18)	36	20	(15, 28)	<0.001

^a^According to WHO Child Growth Standards

^b^the missing eleven were outside the range for the calculation formula

^c^Weight-for-length > 95th percentile according to WHO Child Growth Standards

*BMI* body mass inde, *IQR* interquartile range, *NA* not assessed

In analyses adjusted for potential confounders, AD at enrolment was associated with concurrent excessive weight-for-length (OR 3.03; 95% CI 1.23–7.50) (Table 3) and with concurrent weight-for-length as a continuous variable (OR 1.06; 95% CI 1.04–1.09). Similarly, AD at the two-year follow-up visit was associated with excessive weight-for-length at enrolment (OR 2.40; 95% CI 1.11–5.17) (Table 3). However, AD at enrolment and at two-year follow-up visit was not associated with weight-for-length at birth nor with early weight- gain velocity.

**Table 3:** Number of children with atopic dermatitis (AD) and adjusted odds ratio (OR; 95% CI) for AD at enrolment (mean age 5.1 months) and at two-year follow-up visit (mean age 24.6 months) in 346 children (after excluding children with missing data). The table shows the final models after Hosmer’s stepwise procedure eliminating potential confounding variables^a^

	At enrolment	At two-year follow-up visit
Children with AD, No.	48	100
Excessive weight-for-length^b^ at enrolment	3.03 (1.23–7.50)	2.40 (1.11–5.17)
Parental atopy^c^	4.26 (1.44–12.65)	2.76 (1.43–5.33)
Age^d^	1.28 (1.15–1.41)	1.14 (1.06–1.23)

^a^Weight-for-length at enrolment, age, sex, gestational age, being firstborn child, parental atopy, parental income, parental education, ethnicity, duration of exclusive breastfeeding, duration of breastfeeding, vitamin D levels and recruitment source, i.e. bronchiolitis trial or general population.

^b^Weight-for-length >95^th^ percentile according to WHO Child Growth Standards

^c^Mother and/or father reporting asthma, allergic rhinitis, atopic dermatitis, food allergy and/or urticaria

^d^Continuous variable (months)

In children without AD at enrolment and attending the two-year follow-up visit, weight-for-length as a continous variable at enrolment was associated with an increased risk of AD at the follow-up visit by an OR of 1.02 (95% CI 1.00–1.04) per increase in gram/cm. Atopic dermatitis at the follow-up visit, however, was not associated with concurrent weight-for-length.

Using BMI instead of weight-for-length in all analyses provided similar results. There were no interactions between weight-for-length, BMI, *FLG* mutations and other variables. In infants with AD, AD severity was not associated with weight-for-length.

In children without a *FLG* mutation (*n* = 522), weight-for-length at enrolment increased the risk of AD at the two-year follow-up visit by an OR of 1.03 (95% CI 1.02–1.05), whereas in children with a *FLG* mutation (*n* = 42), the association was statistically non-significant (OR 1.03; 95% CI 0.98–1.09).

Increased TEWL at enrolment, measured in 165 children from the general population only, was associated both with increased risk of concurrent AD (OR 1.07; 95% CI 1.02–1.11) as well as with weight-for-length (Beta 0.04; 95% CI 0.01–0.09).The associations between AD at enrolment and concurrent weight-for-length remained statistically significant when TEWL was included in the model.

## Discussion

In this cohort study with children assessed in infancy (mean age 5.1 months) and at two years of age, AD at both time points was associated with excessive weight-for-length in infancy, but not with excessive weigth-for-length at birth nor with weight-gain velocity from birth to time of examination in infancy.

This study is to our knowledge the first to investigate the role of overweight and weight-gain velocity for the development of AD in the first two years of life. The significant association between AD and excessive weight-for-length in infancy is supported by a study from the UK demonstrating increased wheeze, asthma and eczema in children with high BMI in early childhood [19], and a study from Norway showing association between BMI and atopic sensitization, AD and asthma in later childhood [20]. The lack of associations between AD and weight-for-length at birth is in line with a study in 4-year-old children in Sweden, in which eczema was not associated with weight, length or BMI at birth [21]. Also, in a study among 7-year-old children in Denmark, AD was not associated with increased neonatal size [22]. In contrast, two Danish studies reported that AD in 7-year-old children was associated with birth weight [23], and that AD in the first five years of life was inversely associated with low birth weight [24].

Results from cohort studies on the association between AD and BMI in older children are conflicting, with some studies reporting a positive association [19, 20], some studies reporting no association [25] and one study reporting a negative association [26]. A meta-analysis of studies in children, adolescents and adults concluded that overweight/obesity is associated with an increased prevalence of AD in North American and Asian countries, but not in European countries [2].

An association between AD and excessive weight-for-length (in infants) and overweight/obesity (in children) could be explained by endocrine, metabolic and inflammatory signals from excess adipose tissue affecting other organs, including the skin [27]. Overweight and obesity has been shown to be associated with skin barrier dysfunction and altered immunological responses in children [4, 5]. It has been suggested that obesity results in decreased immunological tolerance to antigens and skewing the immune system towards a Th2 cytokine profile increasing the risk of atopic disease [6]. Other factors, such as dietary, environmental, socio-economic and lifestyle factors, could also play a role [4, 5]. Since AD often starts during infancy and early childhood [3], increased adipose tissue in early life could contribute to the development of AD.

Weight-for-length is often applied for assessing size and weight growth in children younger than 2 years of age [15], while BMI is used as a measure for overweight and obesity in older children, adolescents and adults [8, 28, 29]. Weight-for-length and BMI varies with age, sex and ethnicity [15] and have been shown to be good predictors for obesity and chronic disease in later life [1]. In adults, overweight is defined as BMI >25 kg/m^2^ and obesity as BMI >30 kg/m^2^. The threshold for obesity is not well established for infants [29]. In children, the evaluation of weight, weight-for-length and BMI is often based on WHO-reported growth standards, with weight-for-length >85^th^ percentile and >95^th^ percentile representing larger infants and children [15]. The infants in our cohort were heavier and longer than indicated by these growth standards, which are based on children from several countries, both non-industrialized and industrialized countries, including Norway [15]. The deviation from the WHO growth standards confirms results from other studies showing that more Norwegian children are above the 97.7^th^ percentile (i.e. 2 SD) than expected [30].

In the present study, AD was associated with weight-for-length in infancy in both children with and without *FLG* mutation. FLG deficiency in the skin is known to be a main driver for AD in children with a *FLG* mutation [8]. FLG levels are influenced not only by *FLG* mutation, but also by exogenous stressors and inflammation [31]. In children without a *FLG* mutation, non-mutational mechanisms leading to reduced FLG in the skin must be involved in the development of AD, possibly including factors related to excess weight, as indicated by our findings. It has been shown that obesity is associated with pro-inflammatory cytokines, including tumor necrosis factor-α (TNF-α) [6], which is known to affect FLG levels in the skin [30], and with increased TEWL [4].

In subgroup analysis of the infants with TEWL measurements at enrolment, AD was associated with weight-for-length in infancy even when TEWL was retained in the final models. Although based on a limited number of infants and on TEWL measurements performed with a wider humidity range than in most other studies (11), this is in line with other studies showing skin barrier dysfunction to be associated with overweight and obesity in children [4] and adults [5].

The strengths of the present study include having a cohort of infants living in the same geographical area and recruited from a clinical trial on bronchiolitis and the general population, and a reasonable follow-up rate at 2 years. We find it unlikely that the difference in follow-up rates between the two groups have had any significant impact on the study’s ability to detect associations. Being recruited from the bronchiolitis trial or the general population (i.e. recruitment source) was included in all multiple regression analyses and did not reach the final model in Hosmer’s step down procedure. This indicates that there was no significant effect of recruitment source on the results, despite some heterogeneity between the two groups. All children were examined by experienced physicians, using well-established criteria for AD, as well as reliable measurements of weight and length at all time points. All analyses were adjusted for possible confounding variables. We did not have access to data on maternal health such as weight and/or BMI, which is known to have an impact on the infants’ weight-for-length. Power calculations were performed post hoc. For some subgroup analyses the power is low due to a low number of subjects. Also, multiple statistical analyses increase the risk for type 1 error.

## Conclusion

Our results suggest that overweight may be a contributing factor for the development of AD in early life, highlighting the need for child health-care professionals to address potential overweight and atopic disease when advising infants’ caregivers.

## Abbreviations

AD: Atopic dermatitis
BMI: Body mass index
CI: Confidence intervals
FLG: Filaggrin
SCORAD: SCORing Atopic Dermatitis
SD: Standard deviation
TEWL: Transepidermal water loss
TNF-α: Tumor necrosis factor-α
WHO: World Health Organization
